# GPCR Genes as Activators of Surface Colonization Pathways in a Model Marine Diatom

**DOI:** 10.1016/j.isci.2020.101424

**Published:** 2020-07-30

**Authors:** Weiqi Fu, Amphun Chaiboonchoe, Bushra Dohai, Mehar Sultana, Kristos Baffour, Amnah Alzahmi, James Weston, Dina Al Khairy, Sarah Daakour, Ashish Jaiswal, David R. Nelson, Alexandra Mystikou, Sigurdur Brynjolfsson, Kourosh Salehi-Ashtiani

**Affiliations:** 1 Laboratory of Algal, Systems, and Synthetic Biology (LASSB), Division of Science and Math, New York University Abu Dhabi, Abu Dhabi, UAE; 2 Center for Systems Biology and Faculty of Industrial Engineering, Mechanical Engineering and Computer Science, School of Engineering and Natural Sciences, University of Iceland, Reykjavik, Iceland; 3 Center for Genomics and Systems Biology (CGSB), New York University Research Institute, New York University Abu Dhabi, Abu Dhabi, UAE; 4 Core Technology Platforms, New York University Abu Dhabi, Abu Dhabi, UAE; 5 Department of Biology, United Arab Emirates University (UAEU), Al Ain, UAE

**Keywords:** Genetics, Microbiology

## Abstract

Surface colonization allows diatoms, a dominant group of phytoplankton in oceans, to adapt to harsh marine environments while mediating biofoulings to human-made underwater facilities. The regulatory pathways underlying diatom surface colonization, which involves morphotype switching in some species, remain mostly unknown. Here, we describe the identification of 61 signaling genes, including G-protein-coupled receptors (GPCRs) and protein kinases, which are differentially regulated during surface colonization in the model diatom species, *Phaeodactylum tricornutum*. We show that the transformation of *P*. *tricornutum* with constructs expressing individual *GPCR* genes induces cells to adopt the surface colonization morphology. *P*. *tricornutum* cells transformed to express *GPCR1A* display 30% more resistance to UV light exposure than their non-biofouling wild-type counterparts, consistent with increased silicification of cell walls associated with the oval biofouling morphotype. Our results provide a mechanistic definition of morphological shifts during surface colonization and identify candidate target proteins for the screening of eco-friendly, anti-biofouling molecules.

## Highlights

• The model diatom *Phaeodactylum tricornutum* shifts morphology to form biofilms
• G-protein-coupled receptors (GPCRs) can modulate diatom surface colonization
• GPCR1A expression can induce biofouling morphotype and UV resistance
• Identified genes and pathways can serve as targets for anti-biofouling discoveries

## Graphical Abstract

**Figure:**
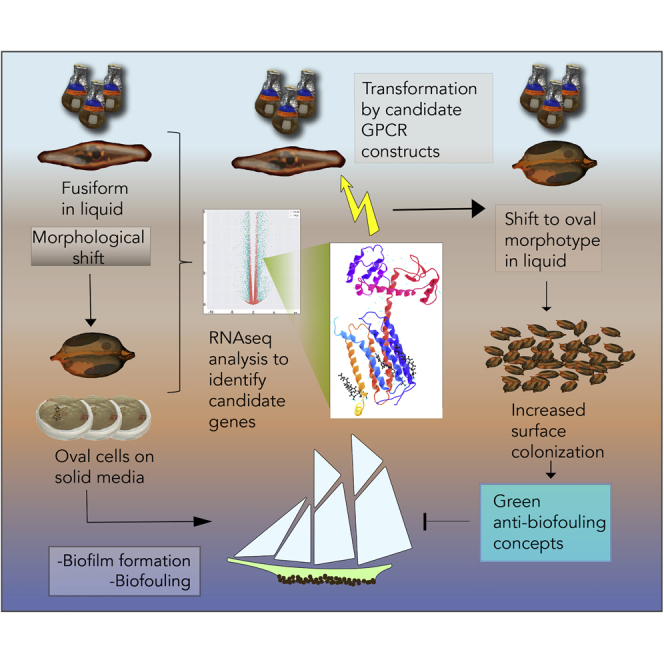

## Introduction

Diatoms (Bacillariophyta) are the dominant group of microalgae in today's oceans (Benoiston et al., 2017; Mock et al., 2017) and one of the most diverse and ecologically important clades of phytoplankton, contributing up to 20% of the global primary production (Bowler et al., 2008; Levitan et al., 2014; Vardi et al., 2008). Diatoms are recognized as the primary contributor to biofilm formation in marine or underwater environments, a process that leads to biofouling (Bruckner et al., 2011; Callow and Callow, 2011; Dang and Lovell, 2016; Finlay et al., 2013; Garacci et al., 2019; Salta et al., 2013; Vanelslander et al., 2012). Marine biofouling has significant impacts on immersed artificial structures such as ship hulls, aquaculture cage facilities, and seawater handling pipes (Leterme et al., 2016; Salta et al., 2013). Particularly, biofouling on ships increases fuel and maintenance costs significantly (Salta et al., 2013). Conversely, surface colonization and biofilm formation have substantial physiological advantages for microalgae in response to stress conditions and also play important roles in microbial adaptation to fluctuations in marine environments (Dang and Lovell, 2016; Schaum, 2019).

A hallmark of the diatom cell wall is its siliceous structure, called the frustule, which is typically made of silicon dioxide (SiO_2_) particles having diameter ranging from 10 to 100 nm (De Tommasi et al., 2017; Vardi et al., 2008). Unlike other known diatoms, the diatom *Phaeodactylum tricornutum*, can grow in the absence of silicon and exists in different morphotypes such as fusiform, oval, and triradiate cell forms. Among these forms, only the oval cells make silicified frustules (De Martino et al., 2011; Lewin et al., 1958). The oval cell forms only become dominant in *P*. *tricornutum* culture population in response to environmental stress and during biofilm formation on solid surfaces in which cells form aggregates (De Martino et al., 2011; Francius et al., 2008). Silica plays an essential role in cell metabolism in *P*. *tricornutum* when it makes morphology shift from fusiform to oval in response to environmental stress. To date, *P*. *tricornutum* is the only known diatom species that can grow without silicon, and this feature makes it an ideal model to study cell morphology and how related molecular regulation underlies adaptive capability in marine environments. Although different strains/accessions of *P*. *tricornutum* differ in phenotypes as well as genotypes (De Martino et al., 2007, 2011), it has been reported that only the oval cell morphotype of *P*. *tricornutum* can adhere to surfaces and give rise to colonies to form compact biofilms (Stanley and Callow, 2007).

Diatoms have evolved intricate signaling pathways in response to environmental fluctuations, allowing them to be the dominant clade of microalgae in oceans (Thompson and Coates, 2017). Among the signaling pathways in cell differentiation and morphogenesis (Basson, 2012), G-protein-coupled receptor (GPCR) signaling pathway is highly conserved across eukaryotes and plays an essential role in signal transduction and response to extracellular stimuli (Chan et al., 2015; Cohen et al., 2017; Port et al., 2013). GPCRs, also known as seven-transmembrane domain receptors, are present in most organisms and mediate many vital signaling processes and physiological functions through their interactions with G proteins and downstream effectors (Shaw et al., 2019). Fungal GPCR-mediated signaling and the understanding of receptor-ligand interactions could facilitate the development of receptor-interfering compounds for disease control (Brown et al., 2018). By manipulating the expression levels of the GPCR components, researchers have engineered the yeast *Saccharomyces cerevisiae* and tuned the receptor and reporter to function in a controllable manner to obtain a platform for biosensor applications (Shaw et al., 2019).

**Figure 1:**
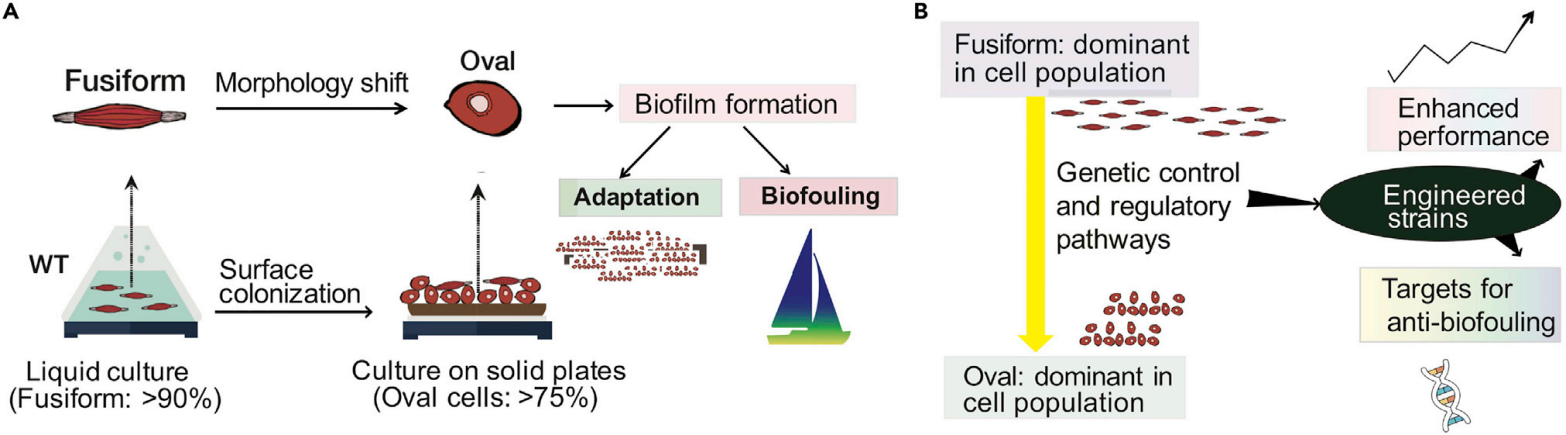
An Overview of the Morphotype Shifting of the Model Diatom *P*. *tricornutum* Associated with Surface Colonization (A) Natural cell morphology shift in diatoms as an indicator for biofilm formation. There are multiple morphotypes in *P*. *tricornutum cultures*; of these, the fusiform cells are dominant in liquid growth under non-stress conditions and the oval cells dominate benthic growth and surface colonization. Fusiform cells gradually shift to oval cells during surface colonization or when subjected to other environmental stress, before biofilm formation and biofouling. (B) By comparing gene expression profiles of different *P*. *tricornutum* morphotypes, putative regulatory pathways and gene products can be identified. The role of these candidate effectors can be validated by their expression in engineering strains. Once confirmed, the identified gene products can be used as targets in future studies to reverse the natural surface colonization process of diatoms in oceans to combat biofouling.

Here, we interrogate the molecular mechanism underlying the morphological shift in *P*. *tricornutum* (strain Pt1 8.6_F_) in relation to surface colonization (Figure 1). The Pt1 8.6_F_ strain of *P*. *tricornutum* was selected as the model in our study as this strain is known to be an optimal reference model for genetic and genomic studies (Fu et al., 2017), with the ability to shift its morphotypes during surface colonization and biofilm formation, in addition to the availability of its complete genome sequence (Bowler et al., 2008). We identify a set of signaling genes that are differentially regulated during morphotype switching. We demonstrate that engineering the strain to overexpress a *GPCR* gene is sufficient to shift the dominant morphotype from fusiform to oval cells during non-stress growth conditions. The engineered strain also achieves similar photosynthesis performance as its fusiform wild-type (WT) counterpart, and it shows more resistance to UV stress, indicating its potential biotechnological applications.

## Results

### Genome-wide Transcriptome Analysis of Two Different Morphotype Cultures

**Figure 2:**
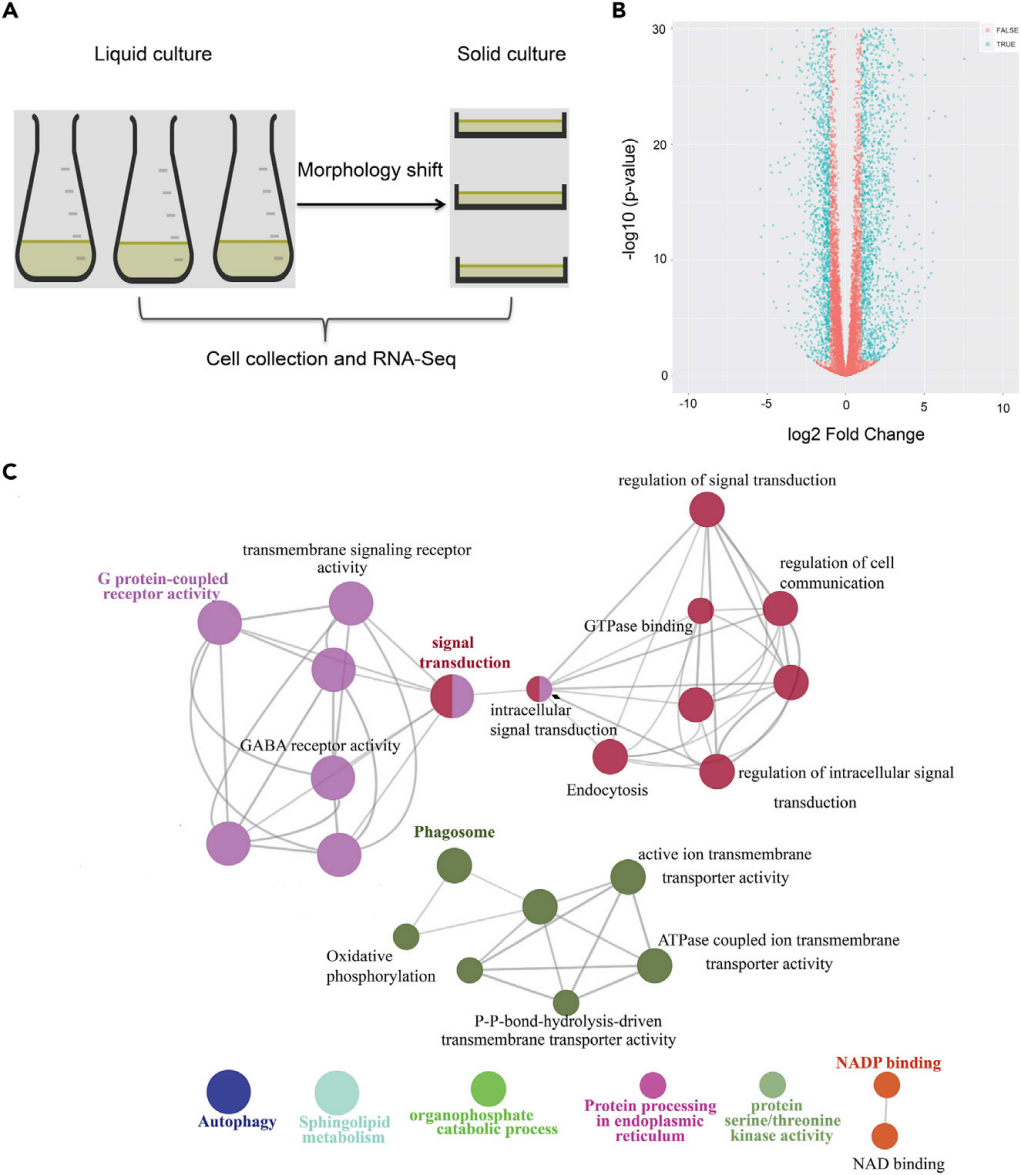
Global Transcriptomic Analysis and Identification of DEGs For a Figure360 author presentation of this figure, see https://doi.org/10.1016/j.isci.2020.101424. (A) Liquid culture (fusiform cells in population >90%) and solid culture (oval cells in population >75%) of the model diatom *P*. *tricornutum* Pt1 8.6_F_ were generated and cells were collected for RNA-seq. (B) Volcano plot with p values and fold changes as log-scaled axes. DEGs are identified with a threshold of log_2_(fold changes) > 1 and a significance level of FDR < 0.05. Significant differences at p value < 0.05 with >2-fold changes are shown in blue color. (C) Gene set enrichment analysis of up-regulated signaling genes highlighting the GPCR signaling pathway. A group of 61 identified signaling genes was used for analysis. GO terms are represented as nodes in the graph, each pathway and its related nodes were presented with a unique color (p < 0.05), and node sizes indicate the relative numbers of genes that represent the GO term, whereas the edges represent genes shared between the GO terms.

To identify transcriptional shift of cells between solid culture (oval > 75%) and liquid culture (fusiform > 75%), we performed RNA sequencing (RNA-seq) on RNA isolated from the WT *P*. *tricornutum* strain Pt1 8.6_F_ grown in liquid and on solid media under low light intensity of 30–50 μmol photons m^−2^ s^−1^ (see Methods). The genome sequence of *P*. *tricornutum* (ASM15095v2 [2013-07-EBI-Phatr3]) was used as a reference to align the transcriptome reads, and normalized counts were calculated to characterize gene expression in cells (see Methods). Among all the expressed genes detected, differentially expressed genes (DEGs) were identified with a threshold of greater than 2-fold and a false discovery rate (FDR) of <0.05. A total of 2,468 genes were up-regulated and 1,878 genes were down-regulated in the solid WT (Figure 2 and Table S1) when compared with the liquid WT cells.

Gene set enrichment analysis (GSEA) (Bindea et al., 2009; Love et al., 2014) was carried out to identify overrepresented Gene Ontology (GO) terms for DEGs from the WT, highlighting cellular biosynthetic processes and signaling-related GO terms in up-regulated genes (Figure S1) and photosynthesis-related GO terms in down-regulated genes (Figure S2). We then performed KEGG Orthology annotations (Figure S3) on a total of 2,468 up-regulated genes from the WT using the KEGG Automatic Annotation Server (http://www.genome.jp/tools/kaas/) and identified 61 genes involved in 44 potential signaling pathways (Figure 2 and Table S2). GSEA for the 61 signaling genes revealed that the GPCR signaling pathway was significantly enriched and five known GPCR-encoding genes, i.e., *GPCR1A* (Phatr3_Jdraft1756), *GPCR1B* (Phatr3_J54411), *GPCR2* (Phatr3_Jdraft1740), *GPCR3* (Phatr3_J44131), and *GPCR4* (Phatr3_J44133), as well as three predicted GPCR genes (Phatr3_J47068, Phatr3_J49346, and Phatr3_J35620) were up-regulated in the solid culture of *P*. *tricornutum* when compared with its liquid culture counterpart (Figure 2). A protein-protein interaction network was also predicted using these 61 signaling genes (59 of 61 were identified in the database) based on the STRING database (Figure S4). We note that the network is fragmented due to the lack of experimental evidence in diatoms.

### GPCR1A Transformants Show Distinct Morphological Shift and Strong Resistance to Light Stress

We queried the KEGG Orthology annotation server with the 2,468 up-regulated genes and identified 61 signaling genes among them. To experimentally interrogate their potential roles in the morphological shift, we synthesized a group of 14 candidate signaling genes with a focus on several GPCR genes because GPCRs are on the top level of signal transduction hierarchy (Figure S5). We expressed these individually in *P*. *tricornutum* cells (Miyahara et al., 2013) with a controllable nitrate reductase promoter (see Methods) to test their ability to shift the cell morphology from fusiform to oval forms. We observed that cells transformed with either a *GPCR1A* or a *GPCR4* construct shifted the population to be dominated by the oval cells.

We conducted surface colonization experiments with these GPCR transformants (i.e., GPCR1A transformants and GPCR4 transformants) on glass slides and found that both transformants showed much stronger attachment and developed more clumps on glass slides over 72 h when compared with their WT counterpart (Figure S6). The oval cell morphotype-dominant transformants were able to colonize surfaces effectively and showed stronger adhesion patterns than their WT counterpart (Figure S6). These results are also in line with those of previous studies on the adhesion strength of different *P*. *tricornutum* species (De Martino et al., 2011; Stanley and Callow, 2007).

**Figure 3:**
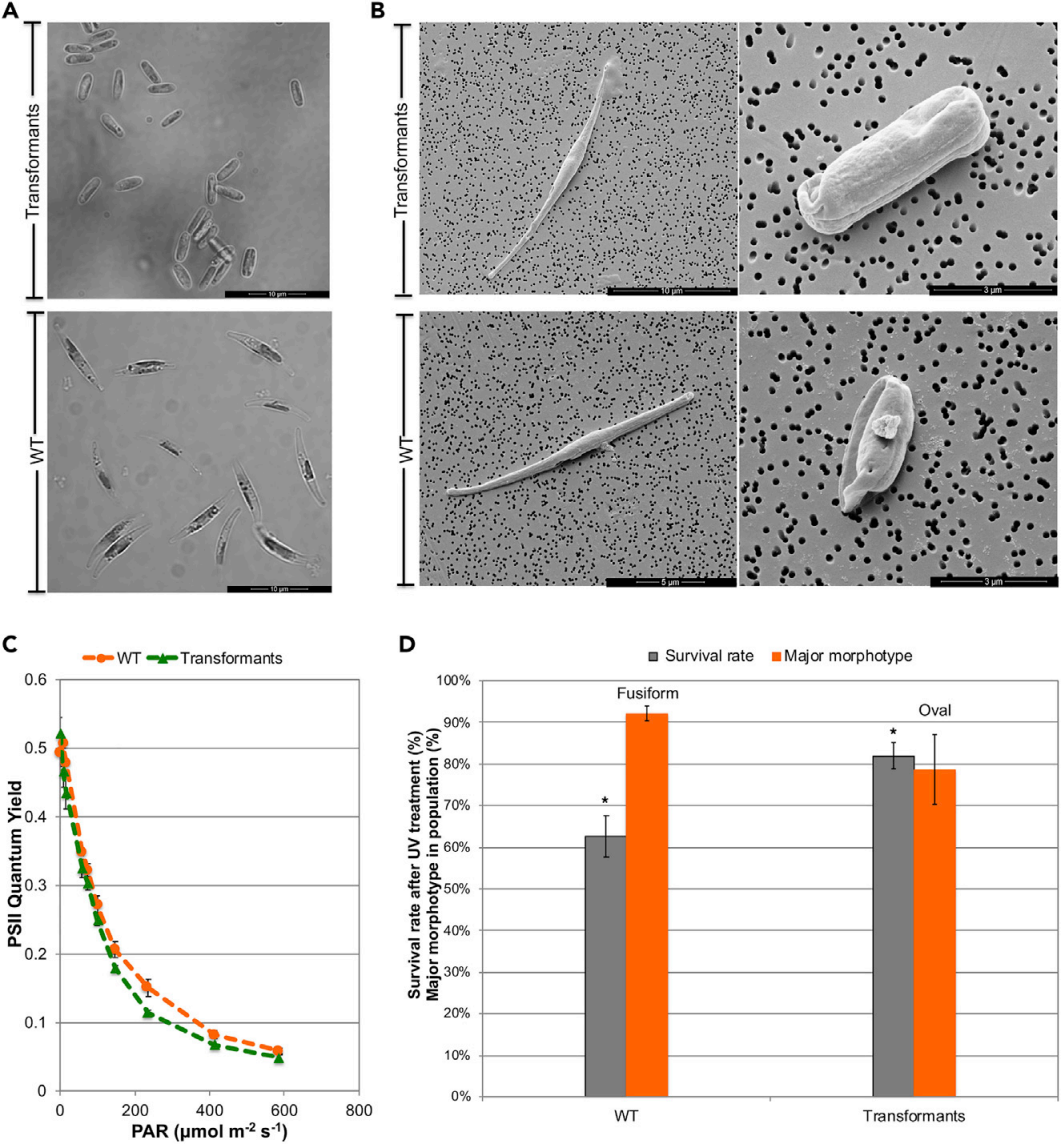
Morphological Shift in the Diatom Population and Its Characteristics (A) Bright-field microscopic images showing the dominant morphotypes in the GPCR1A transformants when compared with the wild-type (WT). (B) Scanning electron microscopic images of different morphotypes in transformants and WT. (C) Determination of the theoretical photosynthetic efficiency (F_v_/F_m_) and effective quantum yield (QY) of photosystem II under a light intensity of 220 μmol photons m^−2^ s^−1^. (D) Enhanced cell resistance to UV radiation in transformants (with oval cells at >75% of the population) when compared with the wild type (with oval cells at <10% of the population). Values were averaged from either two or three independent experiments. WT, wild-type; PAR, photosynthetically active radiation. Error bars indicate SEM. ∗ Indicates statistical significance between the groups (p < 0.05).

The GPCR1A transformants were selected for further experimental investigation. The typical cell morphotypes of the GPCR1A transformants and WT in liquid culture under non-stress growth conditions were observed by light and scanning electron microscopies (Figures 3A and 3B). The average cell size of the GPCR1A transformants (Table S3) in liquid culture was 6.63 ± 0.12 μm, which is similar to that of WT cells on solid culture (6.35 ± 0.12 μm) but significantly larger (p = 0.0016) than those of the cells in liquid culture (5.13 ± 0.25 μm). The transformed oval cells were morphologically similar to the WT oval cells.

To evaluate the strain capability in response to immediate light stress, we determined the effective quantum yields (QY) of photosystem II (PSII) of the transformants using the WT as controls under light stress of 220 μmol photons m^−2^ s^−1^ (Figure 3C). The effective QYs of the transformants were similar to those of the WT of liquid culture (Figure 3C). Moreover, the effects of UV treatment on different strains were studied (Figure 3D) to assess the role of diatom frustules in UV protection as silica cell walls in diatoms can protect DNA from UV light damage (Aguirre et al., 2018). As indicated by their different survival rates (p = 0.031), we found that the transformant cultures, with >75% oval cells, were more resistant to UV-C than the WT cultures of over 90% fusiform cells (Figure 3D). This result indicates that the oval cell form could provide an additional layer of protection against UV irradiation damage.

### Comparative Transcriptomics and Reconstruction of Signaling Network during Surface Colonization

**Figure 4:**
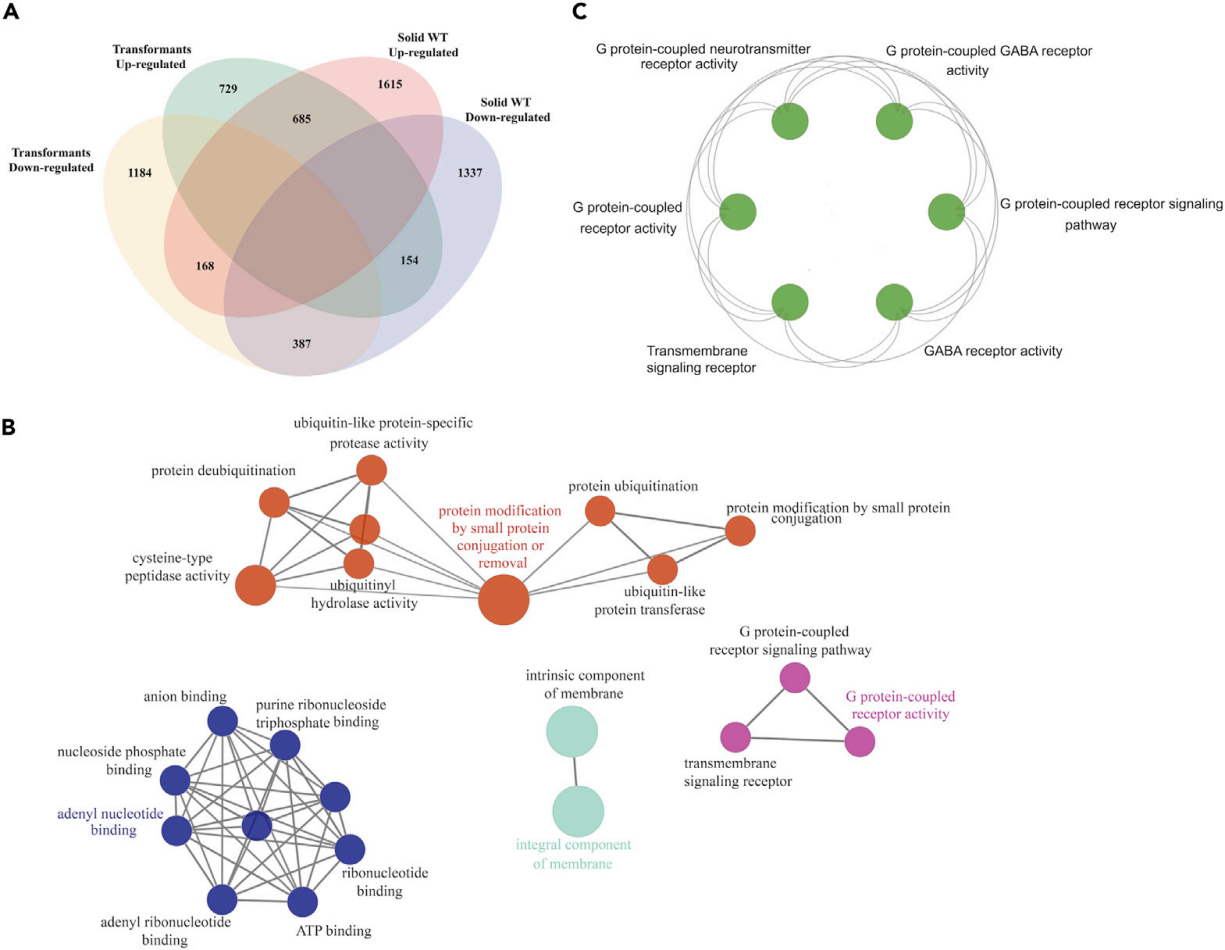
Comparative Analysis of Gene Expression Profiles upon Morphological Shift in Engineered and Wild-Type Strains (A) Venn diagram of shared and unique genes between transformants-derived and solid culture condition-derived DEGs. (B) Gene ontology analysis of the shared, up-regulated DEGs showing the significantly enriched (p < 0.05) terms shared between transformants-derived and solid culture condition-derived morphological shift from fusiform to oval cells. (C) Gene ontology analysis of the signaling genes (a subset of up-regulated DEGs in the transformants) highlighting the significant enrichment of GPCR-related signaling pathways.

**Figure 5:**
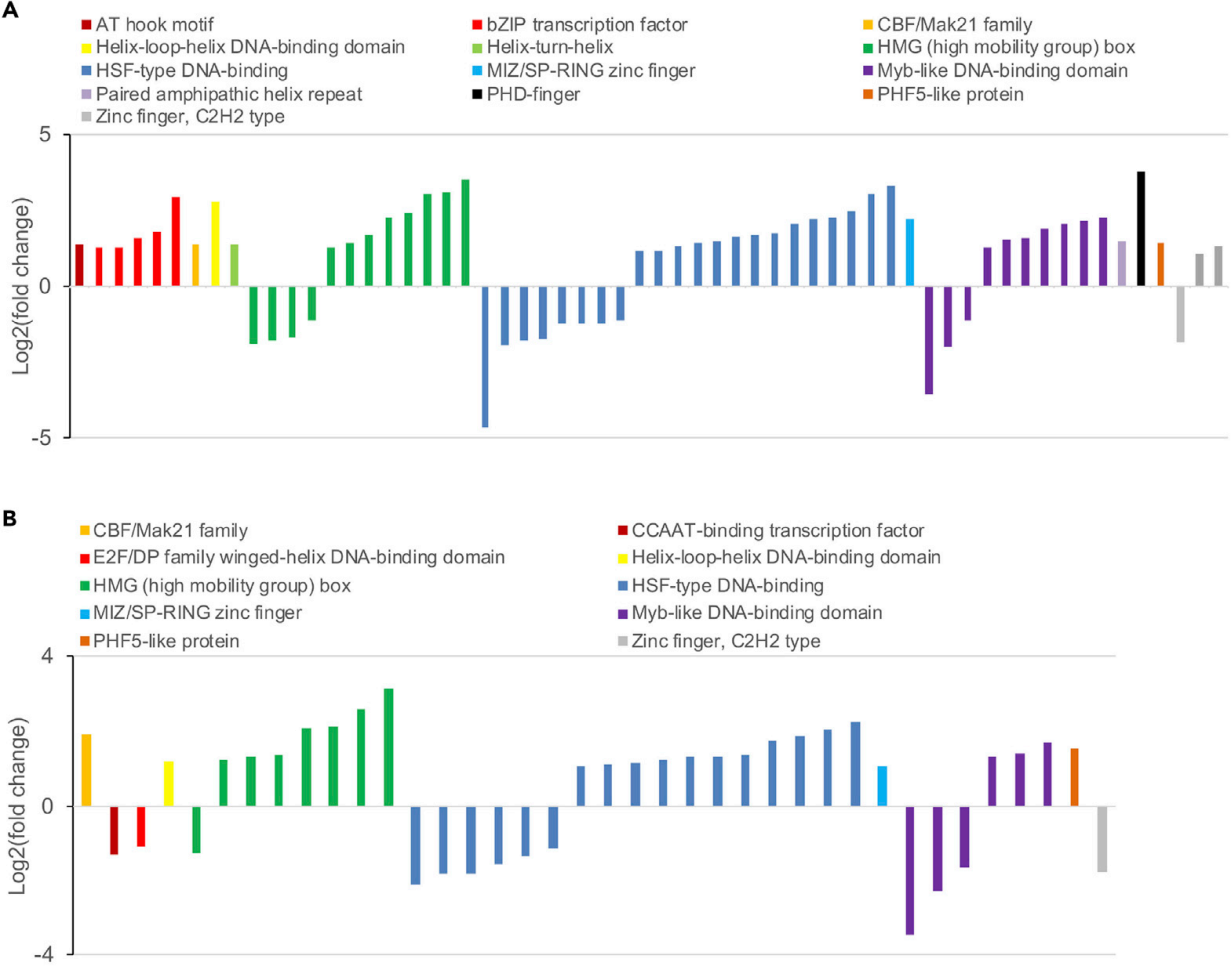
Differential Expression of Transcription Factors (A and B) The expression of transcription factors (TFs) was compared between wild-type (WT) cells grown on solid medium and in liquid (A) and between GPCR1A transformants grown in liquid culture and WT cells grown in liquid (B). The bar graphs represent TF families and their genes with their expression level shown as log_2_ of their fold change. TF annotations for the model diatom *P*. *tricornutum* were obtained from https://phycocosm.jgi.doe.gov/mycocosm/annotations/browser/transfactor/summary;zEFxC9?p=Phatr2.

The GPCR1A transformants displayed oval cells as the dominant form in the population when compared with their WT counterparts under the same non-stress growth condition (Figure S6). The transcriptome analysis of GPCR1A transformants identified 1,568 up-regulated genes and 1739 down-regulated genes when compared with its WT counterpart in liquid culture (Table S4). The DEGs are fewer in the transformants than in the solid WT. Furthermore, 685 of the 1,568 up-regulated genes in the transformants were shared with the identified up-regulated DEGs in the solid WT during the surface colonization process (Figure 4). Among the 685 up-regulated DEGs, four GPCR genes including *GPCR1A*, *GPCR1B*, *GPCR3*, and *GPCR4* were highly expressed in the transformants when compared with their WT counterpart in the liquid culture, showing a similar signaling pattern as the solid WT growing on agar plates (Figure 4 and Table S4). However, the fold changes of the top 100 up-regulated genes from the solid WT, ranging from 21.0 to 279.8 with a median level of 36.2, were much higher than those from transformants, ranging from 8.3 to 143.7 with a median level of 12.1 (Table S5). In addition, 14 of 16 shared genes of the two top-100 lists (Table S5) were at much greater fold changes in the solid WT, indicating that the expression shift was moderate in the transformants compared with those in the condition of harsh environmental stress (e.g., surface colonization). As a consequence of GPCR1A overexpression, downstream effector and regulator genes, including a GTPase-binding protein gene (Phatr3_J26387) and a protein kinase C gene (Phatr3_J14202), were also found to be up-regulated in the transformants. In addition to the GPCR signaling genes and their downstream effectors, we analyzed the transcription factors (TFs) from the two groups of DEGs, i.e., GPCR1A transformants and the solid WT, when compared with the liquid WT (Figure 5). Although many TF families showed both up- and down-expression pattern, one TF, PHF5-like protein (PHD finger-like domain-containing protein 5A), was up-regulated both in the solid culture and in the transformants, when compared with the liquid WT. We note that PHF5-like proteins are reported with a role in cell cycle progression in yeast and morphological development in worms (Oltra et al., 2004).

**Figure 6:**
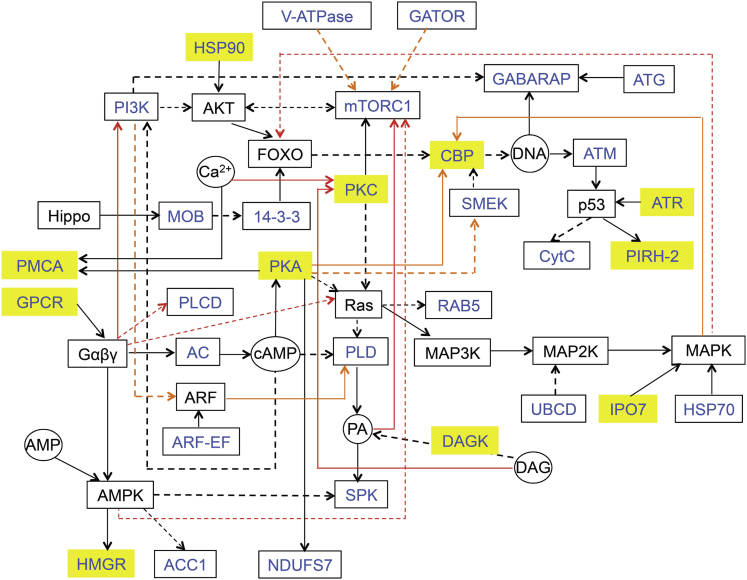
A Reconstructed Putative Signaling Network Involved in Surface Colonization The *P*. *tricornutum* signaling network reconstruction was done using information obtained from KAAS (KEGG Automatic Annotation Server), as well as through manual curation. Annotations were obtained by performing BLAST and assigning orthologs by the BBH (bidirectional best hit) method, against the manually curated KEGG GENES database. Compounds are indicated within circles: AMP, 5′-adenosine monophosphate; Ca^2+^, calcium cation; cAMP, cyclic AMP; DAG, diacylglycerol; PA, phosphatidic acid. Proteins are indicated in square-shaped boxes: 14-3-3, 14-3-3 protein epsilon; AC, adenylate cyclase 1; ACC1, acetyl-CoA carboxylase/biotin carboxylase 1; ARF-EF, ARF guanyl-nucleotide exchange factor; ATG, autophagy-related protein; ATM, serine-protein kinase ATM; ATR, serine/threonine-protein kinase ATR; CBP, E1A/CREB-binding protein; CytC, cytochrome *c*; DAGK, diacylglycerol kinase; GABARAP, GABA(A) receptor-associated protein; GATOR, GATOR complex protein; GPCR, G-protein-coupled receptor; HMGR, hydroxymethylglutaryl-CoA reductase; HSP70, heat shock 70-kDa protein; HSP90, heat shock 90-kDa protein; IPO7, importin-7; MOB, MOB kinase activator; mTORC1, serine/threonine-protein kinase mTOR; NDUFS7, NADH dehydrogenase (ubiquinone) Fe-S protein 7; PI3K, phosphatidylinositol 3-kinase; PIRH-2, RING finger and CHY zinc finger domain-containing protein; PKA, protein kinase A; PKC, protein kinase C; PLCD, phosphatidylinositol phospholipase C, delta; PLD, phospholipase D1/2; PMCA, P-type Ca^2+^ transporter type 2B; SMEK, protein phosphatase 4 regulatory subunit 3; SPK, sphingosine kinase; UBCD, ubiquitin-conjugating enzyme E2 D; V-ATPase, V-type H+ -transporting ATPase. Detailed information on differentially expressed genes is included in Table S6. Solid-line arrows represented direct transduction between two proteins/compounds, whereas dashed-line arrows indicated multiple steps of transduction between two proteins/compounds. The coloring of the lines was done to enhance the diagram. Blue-colored text indicated that the genes encoding the proteins were up-regulated in solid wild-type (WT) culture compared with liquid WT culture, yellow-colored boxes represented the genes encoding the proteins that were up-regulated in liquid transformants culture compared with liquid WT culture; the gene expression information is based on whole-transcriptome analysis carried out in this study.

We reconstructed and manually curated the proposed GPCR-mediated signaling pathway based on the integration of up-regulated DEGs in solid culture when compared with liquid culture information using KAAS (KEGG Automatic Annotation Server, Tables S1, S2, and S6). The proposed pathways included major up-regulated signaling genes as a consequence of growth on solid media (Figure 6 and Table S6). Key effectors for the surface colonization process, including AMPK, cAMP, FOXO, MAPK, and mTOR signaling pathways and their related kinases and activators, are proposed (Figure 6). Among the 32 up-regulated signaling genes in the solid WT when compared with the liquid WT, only 11 signaling genes were found to be up-regulated in the transformants compared with their liquid WT counterpart (Figure 6). The transformants appeared capable of shifting the dominant morphotype from fusiform to oval cells with fewer up-regulated signaling genes than in the solid WT (Figure 6 and Table S6). Moreover, the fold changes of four of the GPCR signaling genes, i.e., *GPCR1A*, *GPCR1B*, *GPCR3*, and *GPCR4,* were 2.87 ± 0.07 (n = 3, ±SEM), 2.41 ± 0.11, 2.55 ± 0.04, and 2.86 ± 0.08 times, respectively, when compared with their liquid WT counterparts; these fold differences were statistically significant (FDR < 0.05). However, the fold change of expression in these GPCR genes in transformants only ranged from 2.41 to 2.87. In contrast, the fold changes for these genes ranged from 4.14 to 49.18 in the solid WT when compared with the liquid WT (Tables S2 and S4), indicating that the expression changes of GPCR signaling genes were more affected by environmental factors.

A schematic wiring diagram of the key signaling components and the downstream targets is illustrated, and the polyamine pathway is highlighted (Figure S7). We note that polyamines play an important role in the promotion of silica precipitation for the formation of siliceous shells of diatoms (Kroger, 2007). Genes encoding well-known cell wall proteins such as silaffins have not been identified in *P*. *tricornutum*; however, the up-regulation of major genes involved in the polyamine pathway alludes to the role of polyamines in the construction of the frustule during oval cell formation (Figure S7).

## Discussion

The model diatom *P*. *tricornutum*, a planktonic species that also has a benthic morphotype (i.e., oval cell form), usually appears as fusiform in liquid cultures under non-stress conditions, and it contributes to biofilm formation and biofouling upon morphology shift and surface colonization (Thompson and Coates, 2017). The cell and molecular biology of biofouling are largely unknown, even though environment-friendly antifouling coatings are urgently needed. In this study, we identified a group of 61 signaling genes involved in *P*. *tricornutum* during the morphological shift of cell populations (Figures 4 and S3). These up-regulated signaling genes include five annotated GPCR genes in diatoms, i.e., *GPCR1A*, *GPCR1B*, *GPCR2*, *GPCR3*, and *GPCR4*. We note that these GPCRs might also be involved in responding to sexual cues in diatoms, such as the planktonic species *Pseudo-nitzschia multistriata* (Basu et al., 2017). In this study, we transformed the WT model diatom species, *P*. *tricornutum*, with GPCR expression constructs, to shift its morphotype and demonstrated that the engineered strain enhanced its resistance to UV light. Moreover, we show that the induced expression of GPCR1A is sufficient to shift morphotypes, pointing to this protein as a potential target protein of screening anti-biofouling compounds. A comparative RNA-seq study on *Phaeodactylum* morphotypes was recently reported on the strain Pt3 (Ovide et al., 2018), in which the major morphotype is oval (60%–75%) under liquid culture conditions. This phenotypic ratio is uncommon and is different in other strains of *P*. *tricornutum*, in which the major morphotype is fusiform (De Martino et al., 2007).

In addition to the observed cell differentiation in diatoms, other eukaryotic microorganisms, such as the yeast, are able to differentiate into specific subpopulations with different characteristics to form organized microbial communities such as biofilms, colonies, and pseudohyphae on solid surfaces with unique properties (Cap et al., 2012). The budding yeast *Saccharomyces cerevisiae* can differentiate from unicellular to pseudohyphal filamentous form in response to stress under the regulation of cyclic AMP-protein kinase A (cAMP/PKA) and the filamentation mitogen-activated protein kinase (fMAPK), rat sarcoma/protein kinase A (RAS/PKA), sucrose nonfermentable (SNF), and target of rapamycin (TOR) signaling pathways (Kim and Rose, 2015). Notably, GPCRs such as Gα subunit Gpa2 of the RAS pathway were recognized during cell differentiation to the filamentous form (Cullen and Sprague, 2012; Pothoulakis and Ellis, 2018). Furthermore, *S*. *cerevisiae* was found capable of forming one colony with two major subpopulations, i.e., U and L cells, occupying the upper and lower colony regions, respectively, and the optimized metabolic properties for U cell long-term existence were activated and controlled by the glutamine-induced TOR pathway amino acid sensor systems (e.g., SPS) together with other signaling pathways under lowered respiration (Cap et al., 2012). Mpk1 (the MAPK) and Bck1 (the MEKK) as well as the receptors that activate and regulate the pathway together with Rlm1 (a TF phosphorylated and activated by Mpk1), all played important roles during yeast cell differentiation into sporulation in colonies (Piccirillo et al., 2015). In this study, PKA and mTOR signaling pathways were also found up-regulated during diatom surface colonization (Figure 6; Tables S1, S4, and S6). Moreover, PKA was up-regulated in the *GPCR1A* transformants when compared with its WT counterpart (Figure 6). These findings highlighted the common function of these signaling components during cell differentiation across the distant phyla of diatoms and yeast.

*P*. *tricornutum* is the only known diatom species that can grow without silicate and has a significant distinction between its two main morphotypes, i.e., fusiform and oval cells, and only oval cells form silicified cell walls (Tesson et al., 2009). With the function of matrix peptides and proteins, silicic acid is converted into amorphous hydrated silicon dioxide with nanostructural properties in diatom cells (Pamirsky and Golokhvast, 2013). However, there is no clear information on the native silaffins in *P*. *tricornutum*, The engineered strain demonstrated enhanced tolerance to UV stress when compared with the WT counterpart, most likely due to the silicified cell walls as diatom SiO_2_ frustules can protect DNA from UV light (Aguirre et al., 2018; Ellegaard et al., 2016). It has also been reported that genetically engineered diatom biosilica can be used for targeted drug delivery (Delalat et al., 2015). These findings may facilitate developing UV stress-resistant strains in industries as well as designing novel applications in biomedicine (Baio et al., 2014).

Marine biofouling has long constituted a major problem for the global maritime and aquaculture industries to alleviate as it has adverse effects and increases operating costs significantly (Callow and Callow, 2011; Salta et al., 2013). The International Maritime Organization banned many traditional biocide-based antifouling coatings, including the very effective and widely used tributyltin (TBT) coatings at the beginning of the twenty-first century due to their high toxicity toward all organisms in the ecosystem and negative impacts on non-target organisms. In this context, an alternative antifouling technology has been in great demand, and much effort has been focused on developing environment-friendly antifouling systems (Magin et al., 2010). Recently, researchers started to explore biomimetic designs for antifouling methods that can combat fouling without toxic side effects (Bixler and Bhushan, 2012; Kang et al., 2016). Understanding the biology of biofouling is important for the development of a successful and robust nontoxic biofouling remedy. As diatoms are the dominant algal group in the ocean and also one of the early colonizers involved particularly in the micro-fouling stage in which biofilm is formed (Dang and Lovell, 2016; Salta et al., 2013), biological understanding of the molecular regulation underlying diatom biofilm formation and dispersal may be beneficial to develop new strategies to control and mitigate biofouling. *P*. *tricornutum* is a polymorphic pennate diatom capable of strong adhesion to the substrates, common in pennate diatoms that all mediate biofouling (Dugdale et al., 2006; Herbstova et al., 2017; Stanley and Callow, 2007). Our findings may pave the way for developing effective and environment-friendly antifouling technology with promising biological targets from signaling pathways. As such, the *GPCR1A* gene identified here is a promising antifouling target. Furthermore, its orthologs in other biofouling organisms can be considered targets to interrogate for developments of a universal, bio-based, antifouling strategy. As morphological shift is a coordinated process during cell development, other regulatory factors such as TFs (Figure 5) may also play important roles and could be of interest for future studies. We expect the novel findings and resources described in this study to serve as a starting point for the discovery of new antifouling targets in the future.

### Limitations of the Study

*P*. *tricornutum* strain Pt1 8.6_F_ was used as an optimal model diatom species in this study as this strain can make a clear shift of morphotype from fusiform to oval cells during surface colonization. We identified and reconstructed a signaling network underlying surface colonization, highlighting the roles of signaling genes, including genes encoding GPCRs. However, the network analysis in this study relied on the reference genome sequence of *P*. *tricornutum* (2013-07-EBI-Phatr3), which is incomplete in functional annotations, as observable in KEGG pathway maps. As a consequence, there might be other key signaling genes and regulatory TFs involved that our study could not identify. In addition, although the analysis of the gene expression of the whole population allowed the identification of DEGs in conjunction with morphological shifts, our RNA-seq analysis looked at asynchronous populations of cells enriched in one or the other morphotype; therefore our reported gene expression changes are limited to measurements of population averages and may not fully reflect the fold changes between individual morphotypes. Surface colonization is a long process (i.e., weeks), and the shift of dominant morphotype in population is a distinctive feature of *P*. *tricornutum* during such process and biofilm formation. There might be transient gene expression events that we likely could not cover with our approach as we only looked at gene expression changes at a single time point; however, our main goal was to identify gene products that could shift cell morphology and maintain it, which we were able to achieve. Finally, the identification of involved signaling genes was based on mRNA expression profiles and not protein levels due to the lack of antibodies for the detection of GPCRs in diatoms. Although we validated the role of *GPCR1A* experimentally, our analysis could have missed other effectors that their activity was modulated, but their expression level at mRNA level was not significantly altered.

### Resource Availability

#### Lead Contact

Further information and requests for resources and reagents should be directed to and will be fulfilled by the Lead Contact, Kourosh Salehi-Ashtiani (ksa3@nyu.edu).

#### Materials Availability

All sequences of synthesized genes can be found in Table S7.

#### Data and Code Availability

RNA-seq data from this article can be found in the GenBank/NCBI data libraries (GenBank: PRJNA566271). The RNA-seq data have also been deposited in Dryad with a unique identifier (https://doi.org/10.5061/dryad.ns1rn8ppx).

## Methods

All methods can be found in the accompanying Transparent Methods supplemental file.

## Supplemental Information

Document S1. Transparent Methods and Figures S1–S8

Table S1. Up-regulated and Down-regulated Genes in Solid WT when Compared with Liquid WT, Related to Figure 2

Table S2. Upregulation of 61 Signaling Genes in Solid WT, Related to Figure 2

Table S3. Measurements of Cell Size in Different Diatom Samples, Related to Figure 3

Table S4. Up- and Down-regulated Genes in Transformants when Compared with Liquid WT, Related to Figure 4

Table S5. Top 100 Up-regulated Genes in Solid WT and the Transformants, Respectively, when Compared with Liquid WT and the Shared Genes of These Top 100 DEGs, Related to Figure 4

Table S6. Detailed Information on These Differentially Expressed Genes, Related to Figure 6

Table S7. Sequences of 14 cDNAs Encoding Candidate Signaling Genes, Related to Figure 6

Figure360. An Author Presentation of Figure 1
