# Supplementary material for: GPCR Genes as Activators of Surface Colonization Pathways in a Model Marine Diatom
Source: iScience. 2020 Jul 30;23(8):101424. doi: 10.1016/j.isci.2020.101424 (PMC7452957; doi:10.1016/j.isci.2020.101424)
Supplement: Document S1. Transparent Methods and Figures S1–S8 [file mmc1.pdf]

iScience, Volume *23*

Supplemental Information

GPCR Genes as Activators of Surface Colonization

Pathways in a Model Marine Diatom

Weiqi Fu, Amphun Chaiboonchoe, Bushra Dohai, Mehar Sultana, Kristos Baffour, Amnah Alzahmi, James Weston, Dina Al Khairy, Sarah Daakour, Ashish Jaiswal, David R. Nelson, Alexandra Mystikou, Sigurdur Brynjolfsson, and Kourosh Salehi-Ashtiani

Supplemental Information

Supplemental Information includes **Transparent Methods**, **eight figures**, and **seven tables** (shown as datasets in Excel files).

Transparent Methods

Algal culture and growth conditions

The strain, *P. tricornutum* Pt1 8.6_F (CCAP 1055/1, Culture Collection of Algae and Protozoa, Scottish Marine Institute, Scotland, UK), was used as a model diatom in this study. The wild type *P. tricornutum* was grown using f/2 +Si medium on either solid agar plates (for surface colonization) or in liquid culture (in flasks) under continuous cool white fluorescent lights of 50 µmol photons m^{-2} s^{-1} at 22° ± 2°C (Fu et al., 2017). Cell quantification was performed by determining the absorbance (at 600 nm) using a spectrophotometer regularly.

Chlorophyll fluorescence measurements for photosynthetic parameters

Chlorophyll fluorescence was measured using a PHYTO-PAM-II (Heinz Walz GmbH). The maximum photosynthetic efficiency of PSII was determined as $F_v/F_m = (F_m - F_o)/F_m$, where F_m is the maximal fluorescence, and F_o is the ground fluorescence in the dark- or low-light adapted cells. The effective quantum yield of PSII in cells was determined as F_v'/F_m' when exposed to the white light of 220 µmol photons m^{-2} s^{-1} over 10 minutes, where F_m' is the maximum fluorescence emission level in the light-acclimated cells. The relative electron transport rate (Cohen et al.) was also obtained for samples during the same experiments.

Generation of genetically modified *P. tricornutum* cells

A group of 14 cDNAs encoding candidate signaling genes (Gene IDs: Phatr3_J22677, Phatr3_J41807, Phatr3_Jdraft1756, Phatr3_J10677, Phatr3_J54411, Phatr3_EG02512, Phatr3_J51511, Phatr3_J12877, Phatr3_J2097, Phatr3_Jdraft1000, Phatr3_J54505, Phatr3_J55230, Phatr3_Jdraft1740, and Phatr3_J44133), including GPCR genes (as shown in Table S7), were synthesized and cloned in the shuttle vector pPha-NR (Fu et

al., 2017) with a nitrate reductase promoter by Twist Bioscience (San Francisco, CA, U.S.A.). The plasmids were then extracted from the bacterial cell cultures using the Qiagen Plasmid Mini kit (Hilden, Germany) after growth and selection in the presence of ampicillin (100 µg/ml). For the transformation of signaling genes in the diatom *P. tricornutum* (Miyahara et al., 2013), the multipulse electroporation protocol was followed as previously described (Fu et al., 2017). PCR verification of the transformants was conducted using genomic DNA and specific primers (**Fig. S8**).

Sample preparation, RNA extraction, and transcriptome sequencing (RNA-seq)

For solid culture experiments of wild type *P. tricornutum*, cells were grown on solid agar (1.2% agar plus the growth medium) for approximately two weeks, while control liquid cultures were grown in flasks for approximately one week (OD_{600}=~0.3). Both culture types were generated using f/2 +Si medium under continuous cool white fluorescent lights of 50 µmol photons m^{-2} s^{-1} at 22° ± 2°C. Cells were collected during the exponential phase for RNA extraction. For each group, three independent experiments were conducted, and triplicates of samples were collected for RNA sequencing studies.

For comparison between the transformed (engineered) *P. tricornutum* and its wild type counterpart, both cultures were grown under the same conditions, in liquid, using f/2 +Si medium in flasks for approximately one week under continuous cool white fluorescent lights of 50 µmol photons m^{-2} s^{-1} at 22° ± 2°C. Cultures were added with 5.0 mM KNO_3 for one day, and cells were collected during the exponential phase for RNA extraction. For each group, three independent experiments were carried out, and triplicate samples were collected for RNA extraction.

Total RNA extraction was performed using the MagMAX-96 Total RNA Isolation kit AM1830 with all necessary reagents (Thermo Fisher Scientific Inc.) according to the manufacturer’s instruction. The complementary DNA (cDNA) libraries were prepared using the TruSeq RNA Library Preparation kit v2 (Illumina) according to the manufacturer’s instruction, as previously described (Fu et al., 2017). After quality inspection, the library was quantified by quantitative PCR for cluster generation on the Bot system and then

sequenced using paired-end sequencing of 2 × 100–base pair read length on an Illumina HiSeq 2500 system (Illumina).

Quantitative comparison of gene expression, and gene set enrichment analysis (GSEA)

The genome sequence of *P. tricornutum* (2013-07-EBI-Phatr3) was used as a reference to align the transcriptome reads (http://protists.ensembl.org/Phaeodactylum_tricornutum/Info/Annotation/). To characterize gene expression in different samples, DESeq2 (Love et al., 2014) was used to identify DEGs between two groups of samples (triplicates within each group). Following the removal of the raw read count <10 in each sample, the total read count was normalized using DESeq2, which scales the read counts by a reference sample based on the geometric mean of the read counts across all samples. RPKM (Reads Per Kilobase of transcript, per Million mapped reads) was used as a normalized unit of transcript expression. Functional analysis and GSEA of DEGs were performed using ClueGO plugin (v2.5.0) in Cytoscape (v3.6.0; (Bindea et al., 2009). ClueGO determined the statistical overrepresentation of GO terms associated with the identified DEGs. The P values were calculated using a two-sided hypergeometric test, and Benjamini-Hochberg false discovery rate (FDR) correction was used to identify the statistical significance of GO terms with corrected $P < 0.05$ for multiple testing. The network was automatically laid out based on the ClueGO visual style, where nodes represented the GO terms, the node size referred to the number of genes in a particular GO, and the color of the nodes reflected the enrichment significance of the GO terms.

Surface colonization on glass slides

Glass microscope slides were first rinsed with deionized water then soaked in 1M HCl for 24 h (Stanley and Callow, 2007). The acid was removed by rinsing the slides using deionized water. The slides were rinsed and dried before adding the prepared diatom culture. The wild type, GPCR1A transformants, and GPCR4 transformants were cultivated under the same condition, then collected during exponential growth. Cells numbers were determined using a hemocytometer under a microscope for all strains. The same amount of cells

(500,000 cells/ml) were used in each experiment. Slides were placed in individual compartments of culture dishes, and 15 ml of cultures at the correct dilution were added. Three replicates were used for each experiment. The cells were allowed to settle for 72 h at 22±2°C in growth chambers, after which the slides were gently shaken/rinsed to remove unattached cells on a shaker at 150 rpm. Cells were then counted from each of the three replicate slides after recording microscope images from 9 randomly sampled views to provide cell adhesion and colonization data.

Statistical analysis

A student's t-test was performed to evaluate the significance of the difference between the two groups of samples for physiology data (p-value < 0.05).

Supplementary figures and figure legends

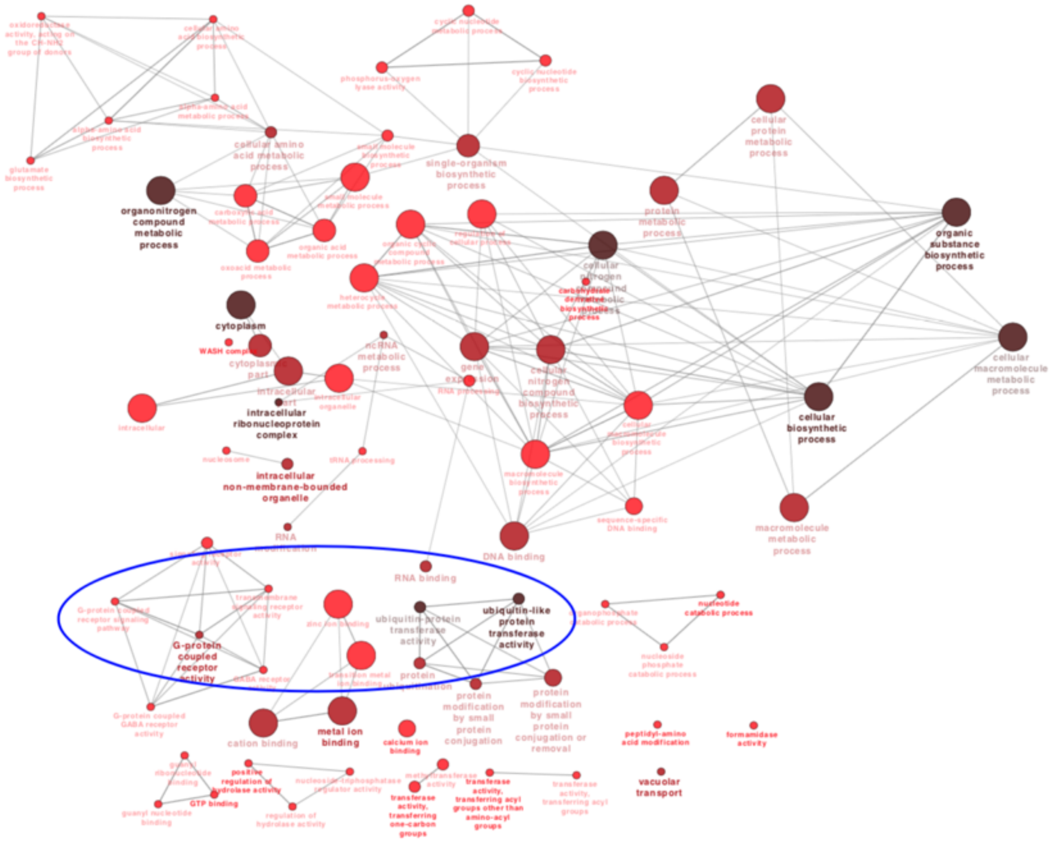

Fig. S1. Gene set enrichment analysis (GSEA) of up-regulated DEGs, related to Figure 2. GSEA was carried out to identify the overrepresented gene ontology (GO) terms for biological process, and molecular function of 2468 identified differentially expressed genes (DEGs) between wild type (WT) strain grown on solid media compared to liquid media growth. Node sizes indicate the relative numbers of genes that represent a GO term; GO terms are represented as nodes in the graph, the color gradient (red to dark red) represents the statistical term enrichment significance (P < 0.05). The blue circle marks the GO terms related to signaling pathways.

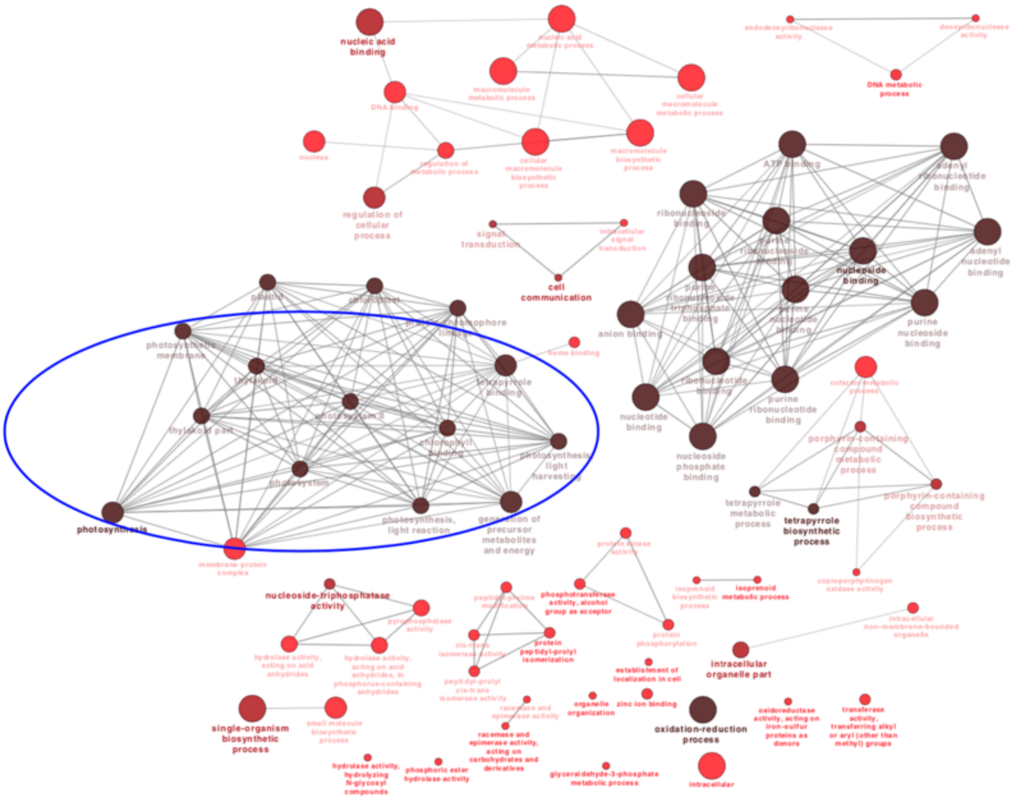

Fig. S2. GSEA of down-regulated DEGs, related to Figure 2. The 1878 DEGs were used to identify overrepresented gene ontology (GO) terms for biological process and molecular function between the growth of WT on solid media in comparison with liquid growth. Node sizes indicate the relative numbers of genes representing a GO term; GO terms are represented as nodes in the graph, the color gradient (red to dark red) represents the statistical term enrichment significance (P < 0.05). Blue circle highlights the GO terms identified in relation to photosynthetic processes.

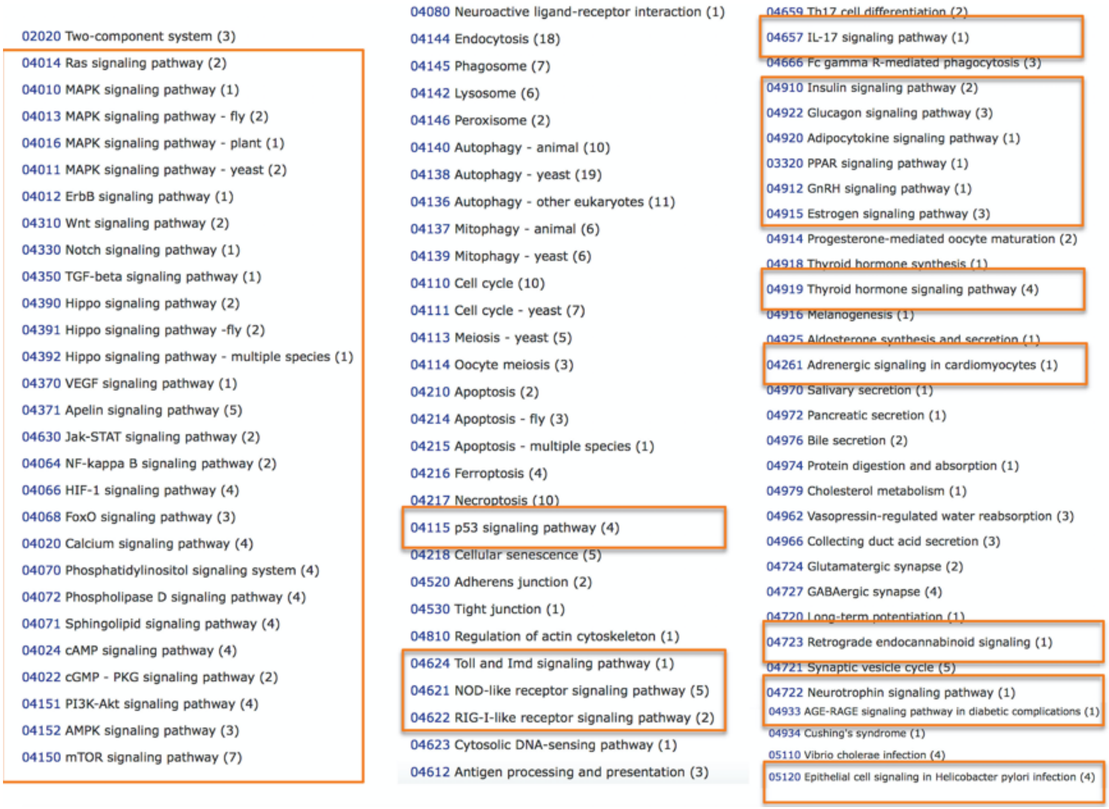

Fig. S3. Identification of signaling pathways and genes using BLAST functional annotation and pathway mapping, related to Figure 2. The sequences of the 2468 up-regulated genes in the WT were searched by BLAST against the manually curated KEGG GENES database (https://www.genome.jp/kegg/kaas/) with a complete genome of *P. tricornutum* using BBH (bi-directional best hit of nucleotide by its default setup) to assign orthologs and provide functional annotation and pathway mapping through KEGG Automatic Annotation Server. The signaling pathways are marked with red boxes. Detailed gene IDs of the 61 identified signaling genes are described in Supplementary Table 2.

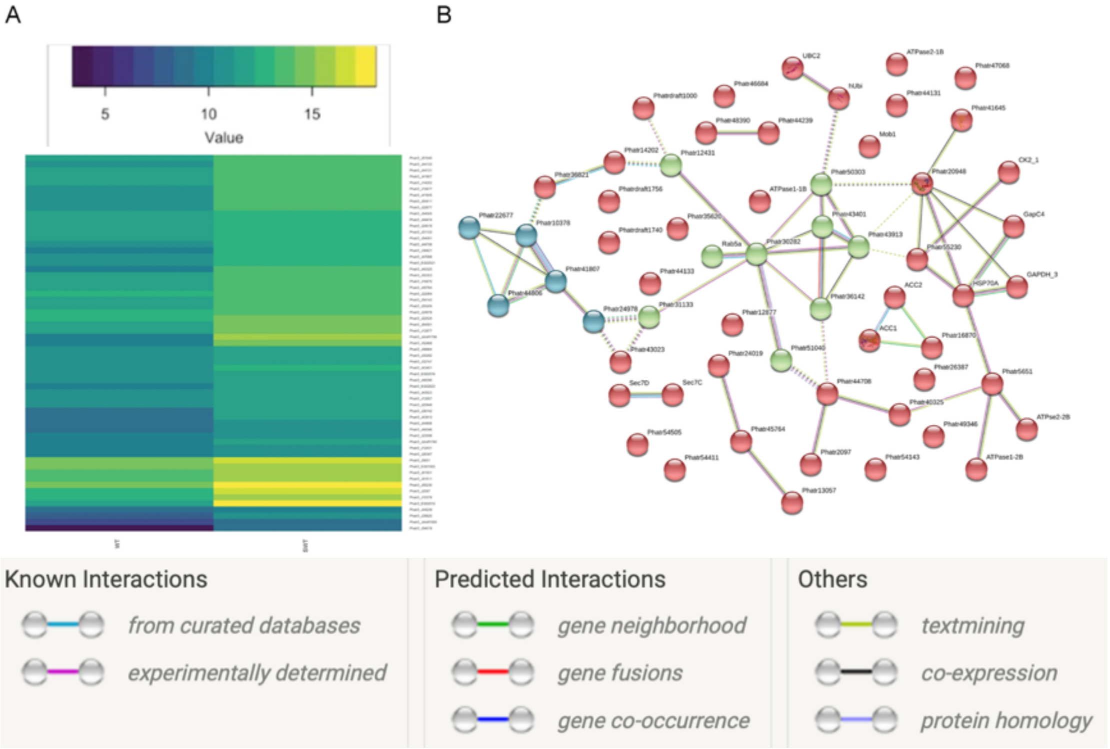

Fig. S4. Gene expression profile of 61 identified signaling genes, related to Figure 3. (A) A heatmap of the identified up-regulated signaling genes in the wild type. The color key represents the value of $\log_2$ (Normalized counts) of RPKM values. **(B)** Reconstruction of gene association network using the up-regulated signaling genes using the STRING database (https://string-db.org). The network is grouped using k-means clustering (with k=3). Nodes represent genes, and node colors represent the k-mean clustering; edge (link) colors indicate the type of evidence for the interactions. Known interactions, predicted interactions, and other interactions are indicated in the lower panel of the figure.

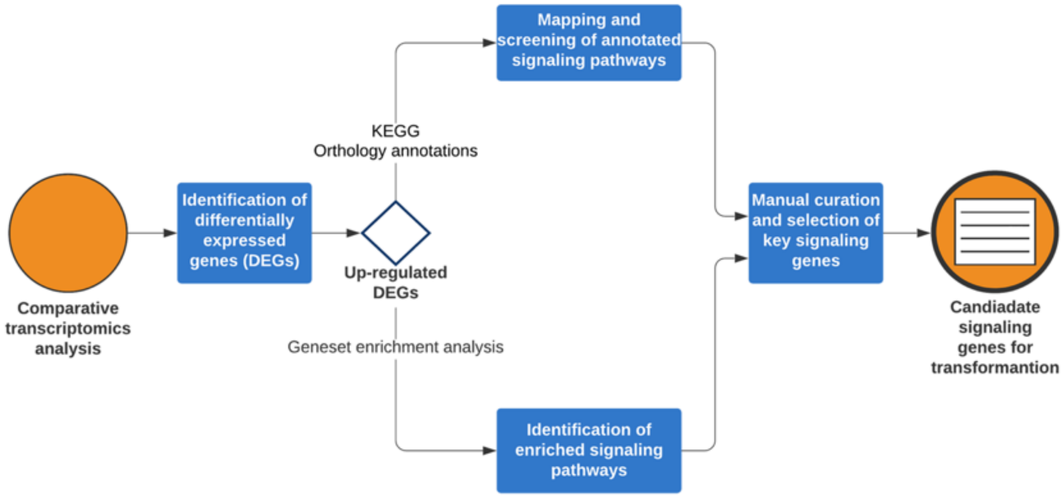

Figure S5. A workflow for screening and selection of candidate genes for overexpression in *P. tricornutum*, related to Figure 3. The principal selection criteria are based on the integration of gene expression information with computational enrichment analyses and manual curation of the results, as shown.

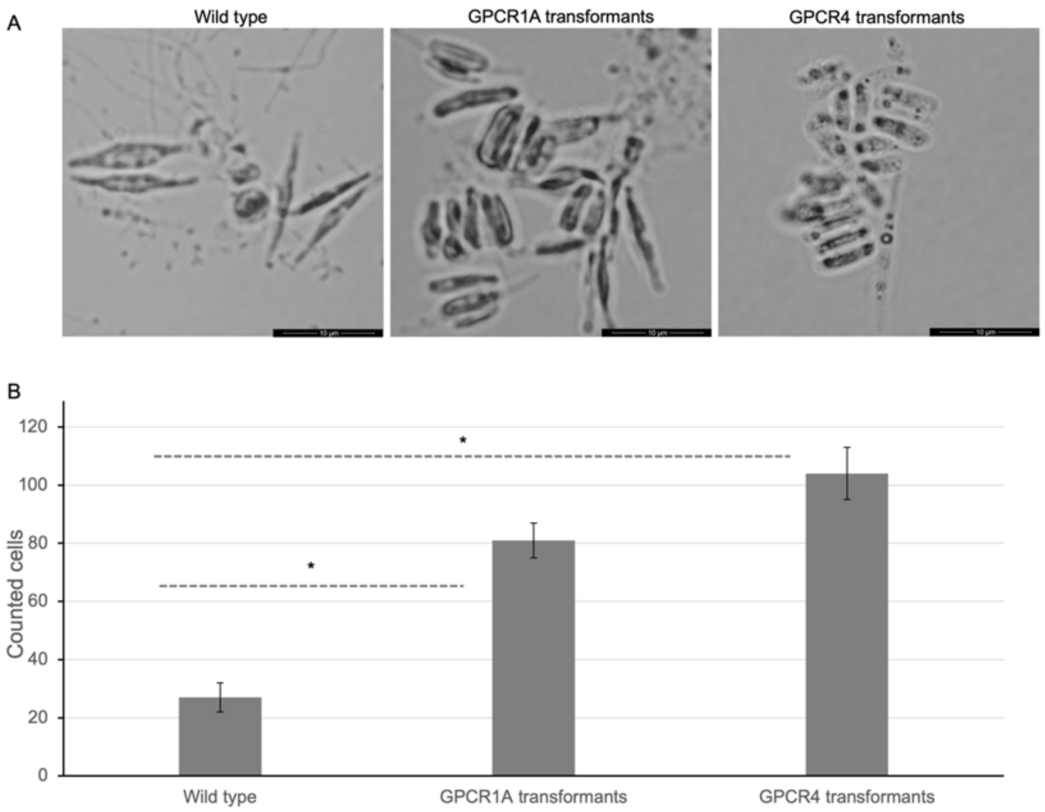

Fig. S6. Surface colonization of the wild type and the two transformants on glass slides over 72 hours, related to Figures 3 and 4. (A) Brightfield microscopy image of the cells on glass slides. Oval cells were observed as the dominant form in the population of the two transformants with the 100X objective lens using oil immersion. **(B)** More cells of transformants were attached to the glass slides in comparison with the wild type. Cells were counted based on a total of 9 fields of view for each glass slide. Detailed information on surface colonization experiments is present in the Methods section. The symbol (*) indicates a significant difference between the two groups ($p=0.032$ for comparison between the wild type and GPCR1A transformants, and $p=0.039$ for comparison between the wild type and GPCR4 transformants, respectively); however, there is no significant difference between GPCR1A transformants and GPCR4 transformants ($p=0.103$).

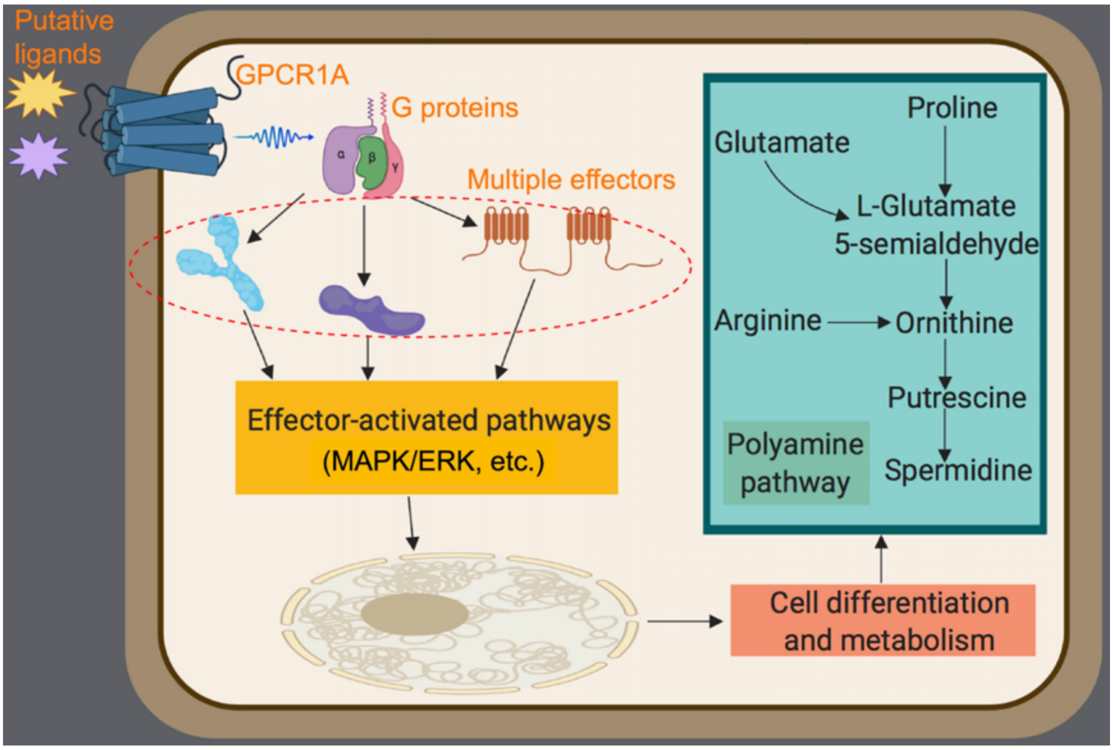

Figure S7. An overview of key putative signaling and metabolic pathways affected in GPCR1A transformants, related to Figure 6. The illustration of major signaling network elements was derived from Figure 6 based on network reconstruction and whole transcriptome analysis. The environmental signals (putative ligands) can bind to GPCR1A, and the associated G-proteins induce the downstream effector-activated pathways (e.g., MAPK/ERK). The G-protein (consisting of α, β, and γ subunits) is separated in the illustration from GPCR1A for the visualization of its activation by the receptor moiety (the drawing does not reflect the actual localization of the G proteins, which are otherwise closely associated with the receptor). The figure also highlights the GPCR-mediated activation of the polyamine synthesis pathway as polyamines play an important role in silica precipitation for the formation of siliceous shells of diatoms. MAPK/ERK: mitogen-activated protein kinase or extracellular signal-regulated kinase.

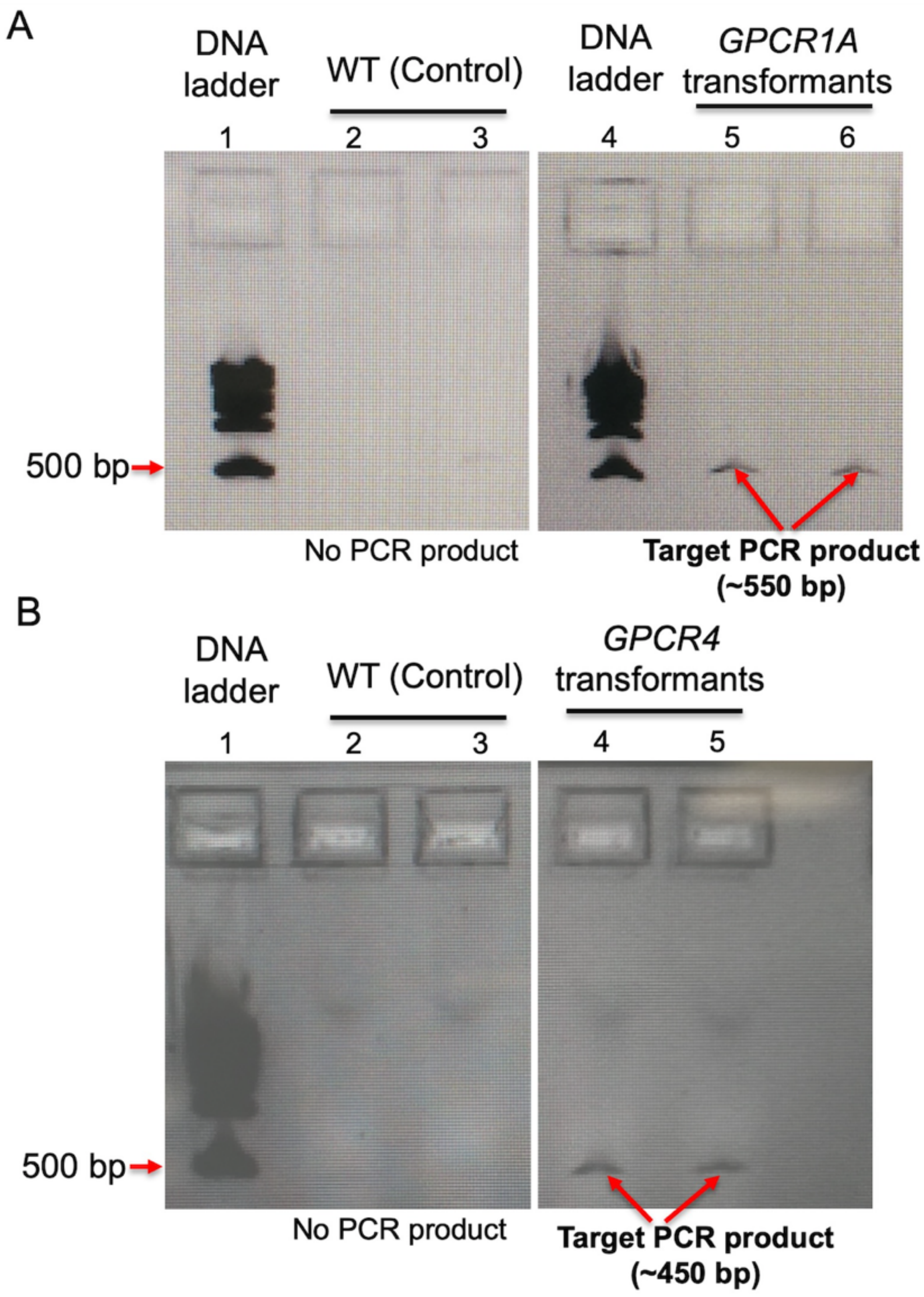

Fig. S8. PCR verification of *GPCR* transformants based on genomic DNA, related to Figures 3 and 4. (A) Primers (Forward, 5’-CACAAACCGAACAGCCCTAC-3’; Reverse, 5’-TCGAGCTTCACAACCTGTCC-3’) were used to amplify DNA fragment across

the nitrate reductase promoter and GPCR1A gene to verify the positive transformation of the synthetic DNA construct into *P. tricornutum* cells; **(B)** Primers (Forward, 5’-CACAAACCGAACAGCCCTAC-3’; Reverse, 5’-CAGTGACGTTGCGACAATCC-3’) were used to amplify DNA fragment across the nitrate reductase promoter and GPCR4 gene to verify the positive transformation of the synthetic DNA construct into *P. tricornutum* cells.
